# Carbapenem-resistant Klebsiella pneumonia infection outbreak in a tertiary urology clinic: analysis of influencing factors with a controlled trial

**DOI:** 10.3906/sag-1909-46

**Published:** 2020-02-13

**Authors:** Fuat KIZILAY, Bayram ALİYEV, Adnan ŞİMŞİR, Serdar KALEMCİ*, Timur KÖSE, Meltem TAŞBAKAN, Hüsnü PULLUKÇU

**Affiliations:** 1 Department of Urology, Faculty of Medicine, Ege University, İzmir Turkey; 2 Department of Biostatistics, Faculty of Medicine, Ege University, İzmir Turkey; 3 Department of Infectious Diseases and Clinical Microbiology, Faculty of Medicine, Ege University, İzmir Turkey

**Keywords:** carbapenem-resistant Klebsiella pneumonia, antibiotic resistance, urological infection, urological surgery

## Abstract

**Background/aim:**

Carbapenem-resistant*Klebsiella pneumoniae* (CR-KP) infections encountered in urology patients differentiate from infections caused by other factors, both in respect to prophylaxis and treatment stage, and require a special approach. We aimed to analyse the predisposing factors and the antibiotherapies for CR-KP infection outbreak in a tertiary urology clinic.

**Materials and methods:**

There were 75 patients in the CR-KP positive group (Group I) and 146 patients in the CR-KP negative group (Group II). Analysis of the predisposing factors for CR-KP infection and comparison of the reinfection rate and the antibiotherapies in the 2 groups were the endpoints.

**Results:**

In the first group, age, comorbidity, previous antibiotic use, and nephrostomy tube rates were higher (P = 0.015, P = 0.001, P = 0.004, and P < 0.001, respectively). In the second group, open urological surgery rate, and the proportion of patients presenting with flank pain, lower urinary tract symptoms, and haematuria were higher (P = 0.029, P < 0.001, P < 0.001, and P = 0.007). In the first group, the proportion of patients treated with transurethral bladder tumour resection was higher, whereas, percutaneous nephrolithotomy was higher in the second group (P = 0.045 for both). While hospitalization and Foley catheterization duration were longer in the first group (P < 0.001 for both), double J stent and nephrostomy duration were longer in the second group (P < 0.001 and P = 0.005). Mean leukocyte count at admission was higher in the first group (P < 0.001).

**Conclusion:**

Advanced age, comorbidities, previous antibiotic use, and prolonged Foley catheterization duration are predisposing factors for this infection in the urology department. Two-week administration of combination antibiotic regimens containing carbapenem were effective for the treatment of this infection.

## 1. Introduction

World-wide increased antibiotic resistance is a global threat to the health system. Recently, carbapenem-resistant gram-negative bacteria have become quite another multiresistant infectious agent with limited treatment options. Carbapenem-resistant *Klebsiella pneumonia* (CR-KP) hydrolyses carbapenems via β-lactamase enzyme and thus shows resistance to many antimicrobial agents [1,2]. Unfortunately, these organisms also have resistance mechanisms for the second line antibiotics. In some recent studies, it has been demonstrated that they may also show resistance to the last option antibiotics such as tigecycline and colistin [3–5]. The importance of antibiotic stewardship in the management of patients has been highlighted and practical guidelines have been developed for the benefit of clinicians for the treatment of urinary tract infections (UTIs) [6,7].

Patients hospitalized in the urology clinic are estimated to have a hospital-related global infection rate of 10%–12% [8]. UTI is the most common type of infection in urology practice, with an annual global case number of approximately 150–250 million [9]. UTIs are a major cause of antibiotic use and resistance due to their high prevalence [10]. However, risks associated with urological procedures can be reduced by appropriate and effective antibiotic regimens.

Patients hospitalized in the urology clinic have a higher rate of urinary catheterization than other clinics and most of these patients undergo urological surgery. On the other hand, urinary catheterization and surgery are well-known risk factors for infection [11]. CR-KP infections encountered in urology patients differentiate from infections caused by other factors, both in prophylaxis and treatment stage, and require a special approach. UTIs caused by CR-KPs are often poor prognostic and difficult to treat [12,13]. 

In the literature, numerous review and meta-analysis are available related to trends in urological infection resistance, their effect on the urological procedures and in regard to antibiotics used. In this controlled trial, we aimed to analyse the factors in the emergence of CR-KP infections in a high-volume urology clinic. 

## 2. Materials and methods

### 2.1. Patient selection and data collection

**Figure 1:**
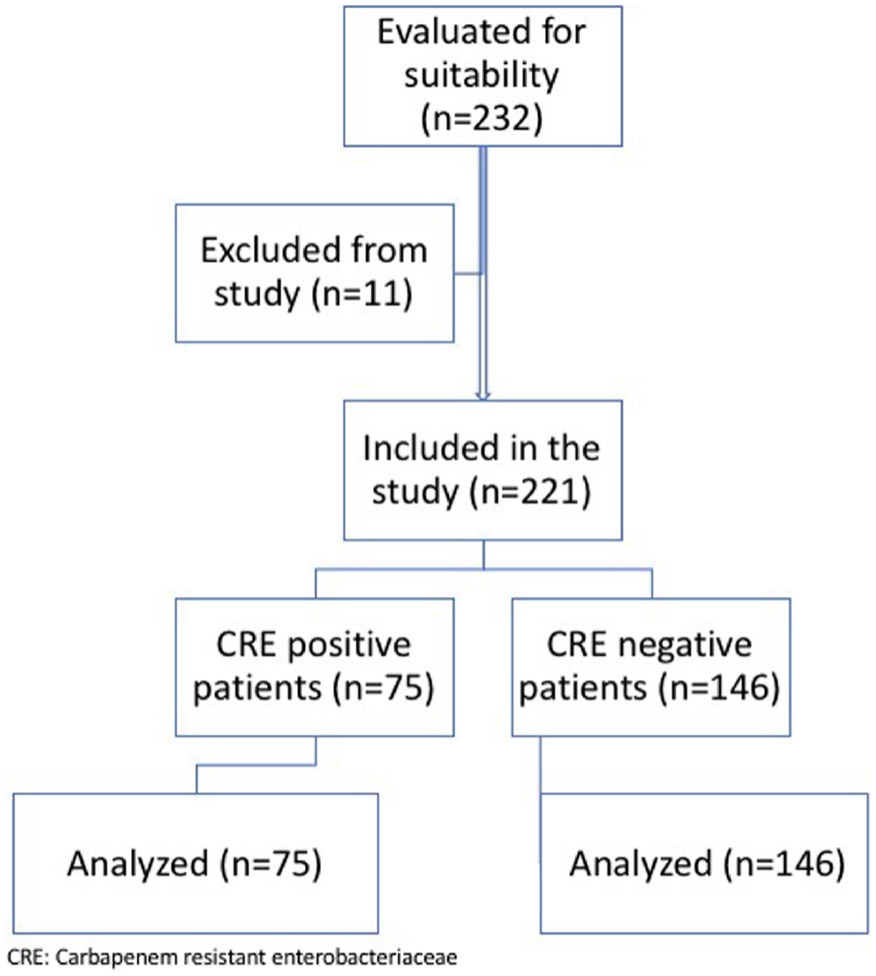
Study flow-chart.

Between June 2016 and January 2019, 81 patients who were treated in our clinic and had a CR-KP in urine culture and 151 patients without CR-KP were the subjects of this study. All patients with CR-KP infection, in that period, were included in the study. The control group consisted of patients who were treated for different reasons in our clinic and had no CR-KP infection in the same period. Six patients whose data were not able to reach in the CR-KP group and 5 patients in the non-CR-KP group were excluded from the study, and the remaining 221 patients were included in the study. The study flowchart is shown in Figure 1.

Demographic data of the patients, operations, duration of hospitalization, presence of a Foley catheter, double J stent and nephrostomy tube, duration of catheterization, complaints at admission, isolated agents in a urine culture, duration of treatment, the antibiotherapies, reinfection rate and treatment of reinfection and isolated agents were recorded from patient files. The data were compared between the 2 groups and the factors that make a significant difference in the development of CR-KP infection among the 2 groups were evaluated. All phases of the study were carried out in accordance with the Declaration of Helsinki or its subsequent amendments. The study protocol was approved by the hospital ethics committee (decision number: 19-5.2T/40 and decision date: 29.05.2019). All patients participating in the study gave written approval for the procedures to be performed. 

### 2.2. Microbiological methods

The minimum inhibitory concentration (MIC) is the lowest antibiotic concentration that inhibits the growth of a particular microorganism for a specific antibiotic. Antimicrobial sensitivity to meropenem and ertapenem is described as follows; resistant, MIC ≥ 16 mg/mL and MIC ≥ 8 mg/L, respectively (for Klebsiella). Sensitivity was defined for colistin, as susceptible, MIC ≤ 0.5 mg/mL (for Klebsiella).

### 2.3. Outcome measures

The primary endpoint of the study was the comparison of predisposing factors between the 2 groups leading to CR-KP infection. The secondary endpoint was a comparison of the reinfection rate and the antibiotherapies in the 2 groups.

### 2.4. Statistical analysis

The means of 2 groups were compared with independent samples t-test. The chi-square tests were used for testing relationships between categorical variables. The pearson chi-square statistic was used in the test of independence. The Mann–Whitney U test is used to compare differences between 2 independent groups when the dependent variable is either ordinal or continuous, but not normally distributed. Logistic regression analysis was performed for variables that were significant in univariate analysis and predictive factors for CR-KP infection development were evaluated. P values that were considered significant in univariate and multivariate analysis were 0.05 and 0.2, respectively. All statistical analysis was performed with the SPSS statistical software program version 22.0 (IBM Corp., Armonk, NY, USA).

## 3. Results

Data of 221 patients were analysed. CR-KP infection was positive in 75 patients, while 146 patients were in the CR-KP negative group. In Group I, the comorbidity rate was higher than Group II (40.0% vs 16.4%, P = 0.001). No significant difference was observed between the 2 groups in terms of proportion of endourologic operations, while the proportion of open operations was higher in Group II (17.3% vs. 32.9%, P = 0.029). Antibiotic use prior to hospitalization was significantly higher in the first group (37.3% vs 16.4%, P = 0.004). There was no significant difference between 2 groups in terms of urological diseases (P > 0.05). In the first group, the rate of private toilet in the hospital room was higher (21.3% vs. 6.8%, P = 0.012). While no significant difference was observed in terms of Foley catheter and double J stent rate, the nephrostomy tube rate was higher in the first group (34.7% vs 4.1%, P < 0.001). Complaints associated with flank pain, lower urinary tract symptoms and haematuria were higher in the second group (2.7% vs 28.8%, P < 0.001; 4.0% vs 27.4%, P < 0.001; 4.0% vs 17.8%, P = 0.007, respectively). Comparison of demographic data, comorbidities and presentation complaints among the groups is summarized in Table 1.

**Table 1:** Comparison of demographic data, comorbidities and complaints at admission of the CRE positive and negative patients.

Variables	Group I(n = 75)	Group II(n = 146)	P-value
Age	64.7 ± 13.2	58.8 ± 15.5	0.015
Comorbidity	30 (40)	24 (16.4)	0.001
Diabetes mellitus	8 (10.7)	10 (6.8)	0.412
Operation history	34 (45.3)	72 (49.3)	0.628
Open urological surgery history	13 (17.3)	48 (32.9)	0.029
Antibiotic use	28 (37.3)	24 (16.4)	0.004
Urological diseases			
Bladder tumour	28 (37.3)	44 (30.1)	0.355
Kidney tumour	5 (6.7)	16 (11)	0.356
Benign prostatic hyperplasia	7 (9.3)	8 (5.5)	0.372
Prostate cancer	15 (20)	18 (12.3)	0.206
Ureteral stone	7 (9.3)	18 (12.3)	0.557
Kidney stone	5 (6.7)	16 (11)	0.356
Urethral stricture	10 (13.3)	18 (12.3)	0.855
Fournier gangrene	2 (2.7)	4 (2.7)	0.978
Hydronephrosis	13 (17.3)	26 (17.8)	0.970
Private toilet in the hospital room	16 (21.3)	10 (6.8)	0.012
Urological catheter			
Foley catheter	65 (86.7)	130 (89)	0.659
Double J stent	28 (37.3)	38 (26)	0.140
Nephrostomy tube	26 (34.7)	6 (4.1)	<0.001
Complaints at admission			
Flank pain	2 (2.7)	42 (28.8)	<0.001
Lower urinary tract symptoms	3 (4)	40 (27.4)	<0.001
Haematuria	3 (4)	26 (17.8)	0.007

Values are given as mean ± standard deviation or number (%) Group I: Carbapenem resistant enterobacteriaceae positive patients; Group II: Carbapenem resistant enterobacteriaceae negative patients. Significant P values are given in bold and italics.

In the first group, the proportion of patients underwent transurethral bladder tumour resection was significantly higher (27% vs 13.7%, P = 0.045), while the proportion of patients underwent percutaneous nephrolithotomy was higher in the second group (2.7% vs 11%, P = 0.045). The comparison of the groups according to the implemented operations is given in Table 2.

**Table 2:** The comparison of the groups according to the operations.

Operation	Group I(n = 75)	Group II(n = 146)	P-value
Endoscopic operations			
Transurethral bladder tumour resection	20 (27)	20 (13.7)	0.045
Ureterorenoscopy	8 (10.7)	16 (11)	0.954
Endoscopic internal urethrotomy	9 (12)	28 (19.2)	0.228
Percutaneous nephrolithotomy	2 (2.7)	16 (11)	0.045
Double J stent implementation	13 (17.3)	20 (13.7)	0.542
Open operations			
Fournier’s gangrene debridement	3 (4)	4 (2.7)	0.671
Radical cystectomy + urinary diversion	6 (8)	4 (2.7)	0.157
Radical nephrectomy	2 (2.7)	10 (6.8)	0.231
Partial nephrectomy	2 (2.7)	6 (4.1)	0.627
Retropubic radical prostatectomy	8 (10.7)	8 (5.5)	0.248

Values are given as number (%) Group I: Carbapenem resistant enterobacteriaceae positive patients; Group II: Carbapenem resistant enterobacteriaceae negative patients. Significant P values are given in bold and italics.

**Figure 2:**
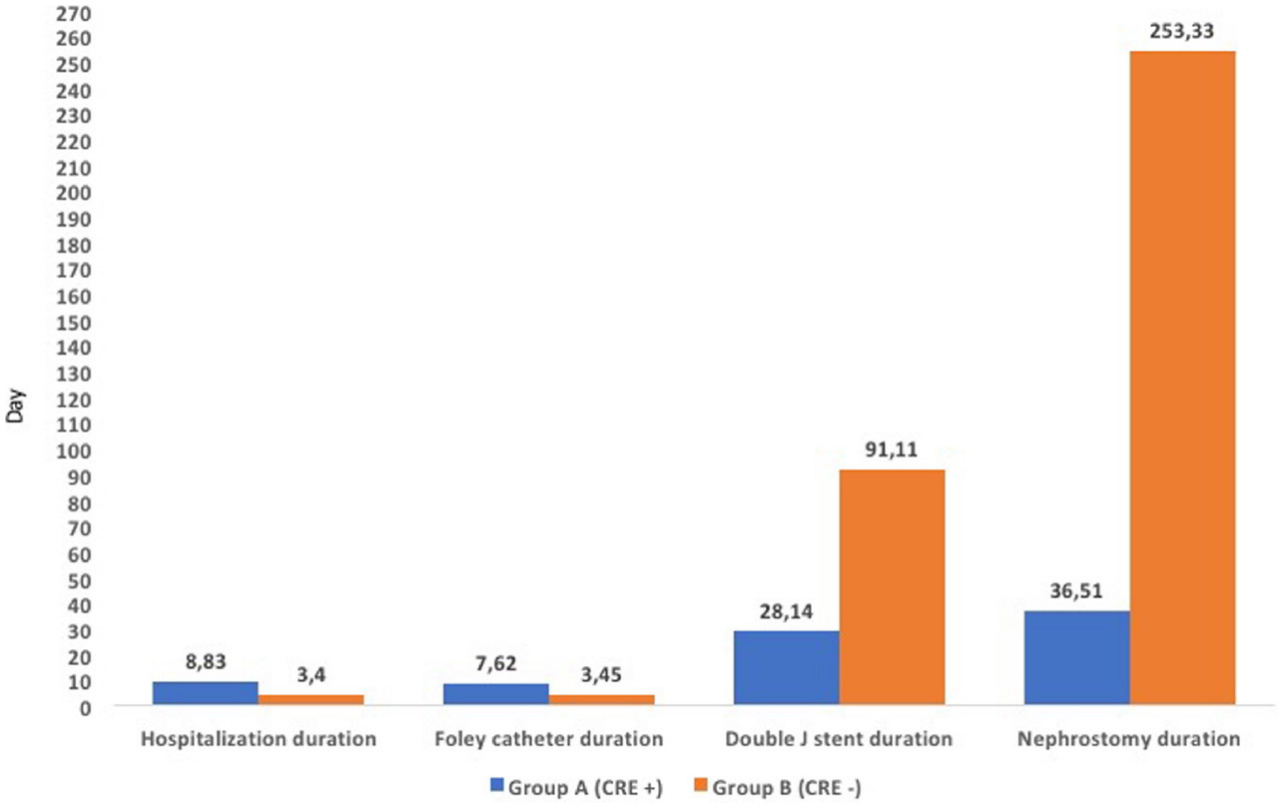
The comparison of hospitalization, foley catheter, double j stent, and nephrostomy tube durations.

There was a significant difference between the 2 groups in terms of hospitalization, Foley catheter, double J stent, and nephrostomy duration (P < 0.001, P < 0.001, P < 0.001, and P = 0.005, respectively). While the mean leukocyte count was significantly higher in the first group (P < 0.001), there was no significant difference between the 2 groups in terms of mean C-reactive protein (CRP) value at admission (P = 0.288). Foley catheter duration was longer in the first group, while the double J stent and nephrostomy tube durations were longer in the second group. The comparison of hospitalization and catheter durations and mean leukocyte and CRP values are shown in Table 3 and Figure 2.

**Table 3:** The comparison of hospitalization and catheter durations and leukocyte and CRP values.

Variables	Group I(n = 75)	Group II(n = 146)	P-value
Hospitalization duration	8.83 ± 9.24	3.40 ± 3.86	<0.001
Foley catheter duration	7.62 ± 7.70	3.45 ± 4.23	<0.001
Double J stent duration	28.14 ± 74.31	91.11 ± 123.24	<0.001
Nephrostomy duration	36.51 ± 93.78	253.33 ± 193.41	0.005
Leukocyte count at admission	10,931.49 ± 5061.90	8211.13 ± 3602.82	<0.001
C-reactive protein at admission	9.67 ± 8.64	5.23 ± 4.84	0.288

Values are given as mean ± standard deviation. Group I: Carbapenem resistant enterobacteriaceae positive patients; Group II: Carbapenem resistant enterobacteriaceae negative patients. Significant P values are given in bold and italics.

**Figure 3:**
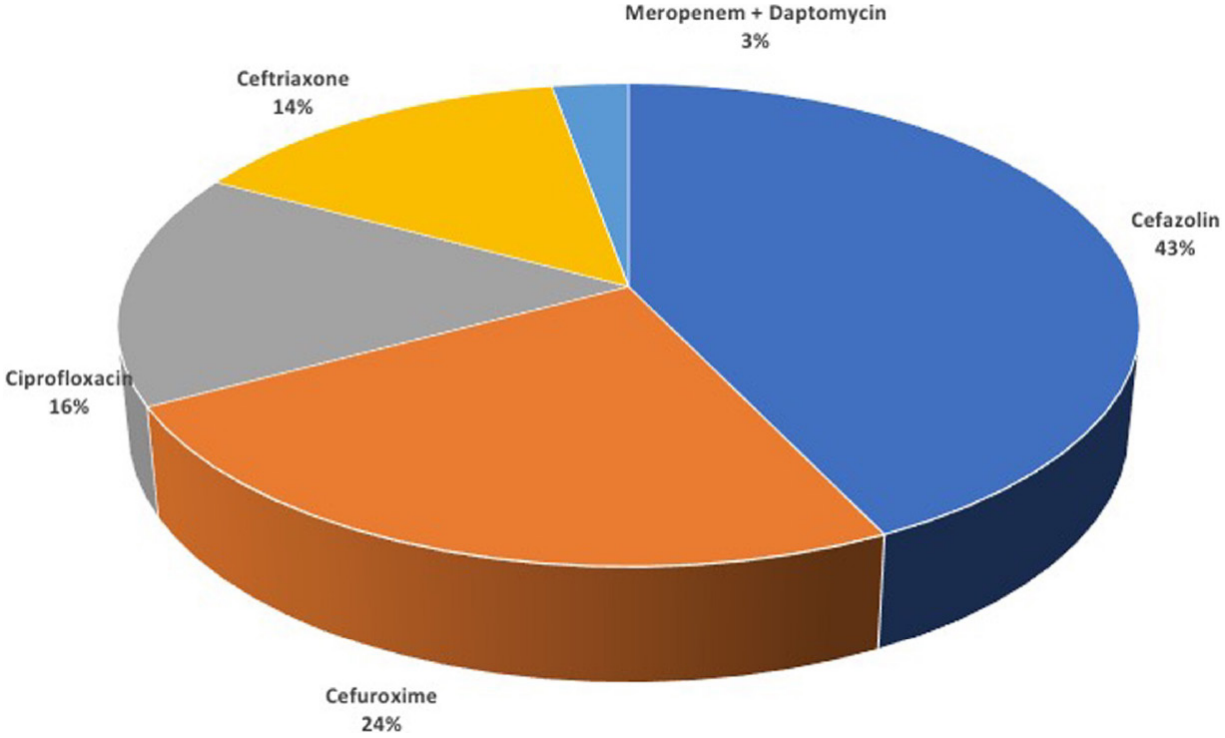
Antibiotics used in CRE negative group.

In the CR-KP negative group, urine culture was positive in 18 patients (12.3%) and 4 of them were *Klebsiella pneumonia* (2.7%). In this group, 140 patients received treatment (95.9%). Antibiotics used in the second group are shown in Figure 3. The most frequently used antibiotic periods were 1-day (20.9%) and 5-day (7.4%) intervals, respectively. None of the patients in this group had a fever of more than 38 °C and reinfection.

**Figure 4:**
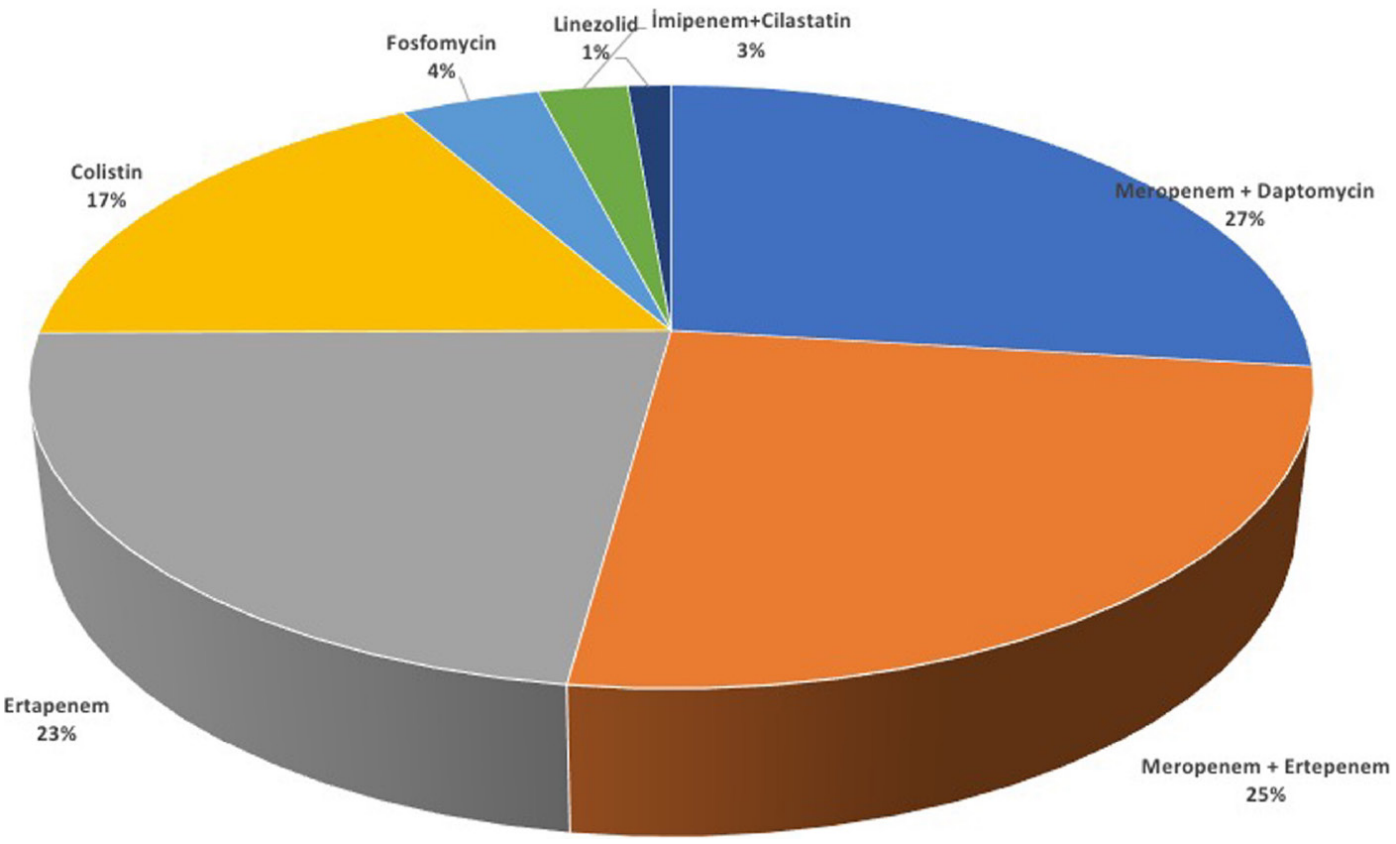
Antibiotics used in CRE positive group.

In the CR-KP positive group, 8 patients presented with fever (10.7%) and 3 patients with sepsis (4%). The urine culture of 74 patients was positive and the isolated agents were CR-KPs in all of them. Treatment was initiated on 80% of the patients (n = 60) and antibiotherapies are summarized in Figure 4. The most common preferred treatments were Meropenem + Daptomycin (26.9%) and Meropenem + Ertepenem (25.2%) combinations. The most common treatment period was 14-day interval in 18.9% of the patients. Reinfection was seen in 20 of these patients (26.7%). In all, the isolated agent in the urine culture was *Klebsiella pneumonia*. Five (25%) had a fever of more than 38 °C. Sixteen (80%) had urological intervention before reinfection and 11 of them were open surgeries (68.7%). Meropenem was used in 49 patients (65.3%) and all of them were drug-resistant (MIC ≥ 16 mg/mL). Ertapenem was used in 35 patients (46.7%) and all of 35 were drug-resistant (MIC ≥ 8 mg/mL). Colistin was used in 14 patients (16.9%) and all were susceptible to the drug (MIC ≤ 0.5 mg/mL).

In the multivariate analysis, age, comorbidity, private toilet in the hospital room, Foley catheter duration, presence of nephrostomy, and complaints on admission were significantly different between the 2 groups. Multivariate analysis of predisposing factors for CR-KP infection is shown in the Table 4.

**Table 4:** Multivariate analysis of predisposing factors for CRE infection.

Variables			95% C.I. for EXP(B)	
	Sig.	Exp(B)	Lower	Upper
Age (years)	0.018	1.029	1.005	1.054
Comorbidity	0.002	3.389	1.565	7.336
Diabetes mellitus	0.416	1.624	0.505	5.217
Endourologic operation history	0.543	1.321	0.539	3.236
Urethral stricture	0.855	1.094	0.417	2.870
Fournier gangrene	0.978	0.973	0.133	7.094
Hydronephrosis	0.970	0.984	0.422	2.295
Fournier gangrene debridement	0.673	1.479	0.240	9.120
Radical cystectomy + urinary diversion	0.176	3.087	0.602	15.822
Private toilet in the hospital room	0.016	3.688	1.274	10.677
Foley catheter	0.659	0.800	0.297	2.156
Foley catheter duration	0.002	1.118	1.043	1.199
Double J stent	0.623	1.215	0.559	2.639
Double J stent duration	0.737	1.001	0.996	1.005
Nephrostomy	<0.001	12.381	3.549	43.197
Nephrostomy duration	0.072	1.005	1.000	1.011
Complaints at admission	<0.001	10.500	4.908	22.462

Significant P values are given in bold and italics.

The most common interval from the urological surgery to CR-KP positivity was a 60-day interval, in 17.9% of the patients, and the second most common interval was 30-day, in 11.9% of the patients. On the other hand, the most common interval from hospitalization to CR-KP positivity was 60-day, in 18.7% of the patients, and secondly 30-day in 10.7% of the patients.

## 4. Discussion

Carbapenem resistance in *Enterobacteria*, which is mainly associated with the mechanisms associated with plasmid-encoded carbapenemases, is an increasing and important global health problem [14–16]. Excessive consumption of carbapenems used to treat extended spectrum beta-lactamase (ESBL) gram-negative infections has led to increased carbapenem resistance, particularly for *E.coli* and *Klebsiella*, which are responsible for the majority of urological infections. However, the significant cost of treatment, prolonged hospitalization and loss of the labour force have emerged.

Patients with indwelling catheters constitute major reservoirs for multi-resistant bacteria. The catheter-associated UTI may be formed by the extraluminal route through the bacteria entering the bladder from the biofilm formed around the catheter in the urethra or intraluminal route by the urinary stasis caused by drainage failure or ascending infection with contamination of the urine collection reservoir [17]. It is known that the duration of catheterization is the most important risk factor for bacteriuria [18]. In patients with indwelling catheter, bacteriuria occurs at a rate of 3%–10% per day of catheter [19]. In our study, we found that patients with Foley catheter and nephrostomy tube had more CR-KP infections and the duration of catheterization was significantly extended in these patients. We could not show a similar effect for the double J stent. Long-term Foley catheterization and nephrostomy tube may be an important risk factor for CR-KP infections by extraluminal route and minimizing the duration of Foley catheter and nephrostomy in these patients may be an effective method to prevent these infections.

High urolithiasis incidence, lower urinary tract symptoms and history of urinary infection, and prolonged use of antibiotics as a result of these are considered as the responsible factors for the development of antibiotic resistance in urological patients [20]. In addition, a patient in the urology clinic has a possibility of having a urological operation of approximately 75%, of which 55% are endourological operations [21]. In non-urological clinics, urinary infections constitute 15%–57% of hospital-acquired infections, while this rate is 70% in urology clinics [21,22]. In our study, there was no significant difference in the prevalence of renal and ureteral stones between the 2 groups. Since our country is located in an endemic region for stone disease and our clinic is a reference centre for stone patients in the region, a large number of stone patients are being treated. Therefore, no significant difference might be observed between the 2 groups. The history of antibiotic use, which is a reflection of previous infections, was found to be higher in the first group, a possible cause of carbapenem resistance. There was also a significant difference between the 2 groups in terms of the operation type, as follows: in the first group, the prevalence of open operation was lower and the prevalence of transurethral bladder tumour resection which is an endourologic operation was higher. Shorter Foley catheter duration in open operations and longer catheterization duration due to resection of large and necrotic bladder tumours and recurrent haematuria attacks may be responsible from this difference.

Age and comorbidities such as immunosuppression, diabetes, obesity, liver dysfunction and malnutrition are well known risk factors for hospital-acquired infections [23]. In our study, we found that the first group consisted of older and more comorbid patients in accordance with this data. 

In areas with high antibiotic resistance, antibiotics such as fosfomycin, nitrofurantoin, tigecycline, colistin, and carbapenem are recommended for the treatment of UTIs rather than fluoroquinolone [24]. Since colistin is nephrotoxic, and it should not be used alone in this infection, because of increasing resistance. The MIC values of microorganisms guide the selection of antibiotics, and preferably the combination with a carbapenem group antibiotic is beneficial. In general, the combination therapy with a carbapenem group antibiotic has been shown to provide a synergistic effect in the treatment of these resistant infections [25,26]. The rationale of combination of 2 or more agents is the reduction in the mortality rate caused by severe CR-KP infections, resistance may occur during monotherapy, and insufficient evidence of the effectiveness of monotherapy. In many observational studies, it has been shown that combination therapy can reduce mortality rates [27,28]. Therefore, although resistant to all, we preferred antibiotic combinations of carbapenem group containing meropenem and ertapenem in 65% and 46% of our patients, respectively. In a retrospective study of 230 patients, ertapenem was shown to be effective in the treatment of complicated UTIs caused by ESBL-producing microorganisms, and in particular, patients undergoing urinary catheterization, urologic intervention and those with diabetes were under risk for superinfection [29]. If the isolate is susceptible, polymyxin-based combination regimens may also be beneficial. In our study, colistin, which was used in approximately one-fifth of patients, was susceptible for all isolates with very low MIC values. However, the transition of tigecycline to urine is 1%, it may also be considered among the second-line treatment agents. Plazomicin is a promising new aminoglycoside antibiotic in the treatment of carbapenemase producing isolates resistant to old aminoglycosides [30]. In our study, dual combination therapies were the most preferred treatment protocols for CR-KP infections and 14-day antibiotherapy interval was the most commonly used treatment protocol. The reason for the high rate of reinfection may be the rehospitalization of these patients due to recurrent endourologic interventions such as control cystoscopy and ureteroscopy.

Empirical treatment can be introduced without any urine culture with a carefully taken clinical history for the treatment of UTIs. However, in some areas with high antibiotic resistance or in cases where symptoms are uncertain, the infectious agent should be identified with microbiological analysis. In our study, we found that the group with negative CR-KP infection was more symptomatic (flank pain and lower urinary tract symptoms) than the positive group. 

Our study has some limitations. Our retrospective study included a short period of time when CR-KP infections peaked. Secondly, this study, which reflects the results of a single-centre, has a relatively limited number of patients. 

In conclusion, CR-KP infection is an increasing problem and necessary precautions should be taken in the urology clinics for prevention and prophylaxis and treatment should be administered with appropriate agents. In particular, advanced age, comorbidities, previous antibiotic use, transurethral resection, long-term Foley catheterization are important risk factors for these infections. Notably, the two-week combination of glycopeptide and lipopeptide antibiotics with carbapenem group antibiotics appears to be an effective treatment method for CR-KP infections. The etiopathogenesis of CR-KP infections in urology patients may be better clarified in controlled-prospective studies conducted with large patient groups.

## Author contributions 

F Kızılay, B Aliyev, A Şimşir, H Pullukçu: Protocol/project development; F Kızılay, B Aliyev, A Şimşir, S Kalemci, M Taşbakan, H Pullukçu: Data collection or management; F Kızılay, T Köse: Data analysis; F Kızılay, H Pullukçu: Manuscript writing/editing.

## Informed consent

Informed consent was obtained from all individual participants included in the study. Authors declared that the research was conducted according to the principles of the World Medical Association Declaration of Helsinki “Ethical Principles for Medical Research Involving Human Subjects” (amended in October 2013). The study protocol was approved by the hospital ethics committee (decision number: 19-5.2T/40 and decision date: 29.05.2019).

## References

[ref0] (2013). Clinical epidemiology of the global expansion of Klebsiella pneumoniae carbapenemases. The Lancet Infectious Diseases.

[ref1] (2016). Epidemiology of carbapenem-resistant Klebsiella pneumoniae colonization: a surveillance study at a Turkish university hospital from 2009 to 2013. Diagnostic Microbiology and Infectious Disease.

[ref2] (2015). Outbreak of colistin-resistant, carbapenemase-producing Klebsiella pneumoniae: Are we at the end of the road. Journal of Clinical Microbiology.

[ref3] (2014). Colistin resistance superimposed to endemic carbapenem-resistant Klebsiella pneumoniae: a rapidly evolving problem in Italy, November 2013 to April 2014. Eurosurveillance.

[ref4] (2014). Tigecycline therapy for carbapenem-resistant Klebsiella pneumoniae (CRKP) bacteriuria leads to tigecycline resistance. Clinical Microbiology and Infection.

[ref5] (2016). Urological infections due to multidrug-resistant bacteria: what we need to know?. Urologia Journal.

[ref6] (2017). The San Luigi Gonzaga Hospital experience: improving blood and urine culture preanalytical quality by shared protocols. Microbiologia Medica.

[ref7] (2007). Prevalence of hospital-acquired urinary tract infections in urology departments. European Urology.

[ref8] (2012). Uropathogenic Escherichia coli mediated urinary tract infection. Current Drug Targets.

[ref9] (2010). Antimicrobial agents for treating uncomplicated urinary tract infection in women. Cochrane Library: Cochrane Reviews.

[ref10] (2004). Nosocomially acquired urinary tract infections in urology departments. Why an international prevalence study is needed in urology. International Journal of Antimicrobial Agents.

[ref11] (2014). Japan Initiative for Global Research Network on Infectious Diseases). Tropical Medicine and Health.

[ref12] (2015). Treatment options for carbapenem-resistant enterobacteriaceae infections. Open Forum Infectious Diseases.

[ref13] (2014). European centre for disease prevention and control. Nursing Standard.

[ref14] (2012). First detection of VIM-4 metallo-beta-lactamase-producing Escherichia coli in Russia. Clinical Microbiology and Infection.

[ref15] (2014). The spread and acquisition of NDM-1: a multifactorial problem. Expert Review of Anti-infective Therapy.

[ref16] (1957). Entry of bacteria into the urinary tracts of patients with inlying catheters. The New England Journal of Medicine.

[ref17] (2010). Diagnosis, prevention, and treatment of catheter-associated urinary tract infection in adults: 2009 International Clinical Practice Guidelines from the Infectious Diseases Society of America. Clinical Infectious Diseases.

[ref18] (2012). Complications of Foley catheters—is infection the greatest risk. Journal of Urology.

[ref19] (2013). An 11-year analysis of the prevalent uropathogens and the changing pattern of Escherichia coli antibiotic resistance in 38,530 community urinary tract infections. Irish Journal of Medical Science.

[ref20] (2017). Prospective study nalysing risk factors and characteristics of healthcare-associated infections in a Urology ward. Investigative and Clinical Urology.

[ref21] (2008). Four country healthcare associated infection prevalence survey 2006: overview of the results. Journal of Hospital Infection.

[ref22] (2014). Saint S. Diagnosis, management, and prevention of catheter-associated urinary tract infections. Infectious Disease Clinics of North America.

[ref23] (2011). Antimicrobials in urogenital infections. International Journal of Antimicrobial Agents.

[ref24] (2018). In vitro and in vivo activity of single and dual antimicrobial agents against KPC-producing Klebsiella pneumoniae. Journal of Antimicrobial Chemotherapy.

[ref25] (2017). In vitro interaction of ceftazidime-avibactam in combination with different antimicrobials against KPC-producing Klebsiella pneumoniae clinical isolates. International Journal of Infectious Diseases.

[ref26] (2017). Effect of appropriate combination therapy on mortality of patients with bloodstream infections due to carbapenemase-producing Enterobacteriaceae (INCREMENT): a retrospective cohort study. The Lancet Infectious Diseases.

[ref27] (2013). Carbapenem-sparing antibiotic regimens for infections caused by Klebsiella pneumoniae carbapenemase-producing K. pneumoniae in intensive care unit. Clinical Infectious Diseases.

[ref28] (2016). Treatment efficacy and superinfection rates in complicated urinarytract infections treated with ertapenem or piperacillin tazobactam. Turkish Journal of Medical Sciences.

